# Germline rare deleterious variant load alters cancer risk, age of onset and tumor characteristics

**DOI:** 10.1038/s41698-023-00354-3

**Published:** 2023-01-27

**Authors:** Myvizhi Esai Selvan, Kenan Onel, Sacha Gnjatic, Robert J. Klein, Zeynep H. Gümüş

**Affiliations:** 1 Department of Genetics and Genomic Sciences, Icahn School of Medicine at Mount Sinai, New York, NY 10029 USA; 2 Center for Thoracic Oncology, Tisch Cancer Institute, Icahn School of Medicine at Mount Sinai, New York, NY 10029 USA; 3 Precision Immunology Institute, Icahn School of Medicine at Mount Sinai, New York, NY 10029 USA; 4 Oncological Sciences, Tisch Cancer Institute, Icahn School of Medicine at Mount Sinai, New York, NY 10029 USA

**Keywords:** Cancer genomics, Cancer genomics, Cancer genomics, Risk factors, Cancer genetics

## Abstract

Recent studies show that rare, deleterious variants (RDVs) in certain genes are critical determinants of heritable cancer risk. To more comprehensively understand RDVs, we performed the largest-to-date germline variant calling analysis in a case-control setting for a multi-cancer association study from whole-exome sequencing data of 20,789 participants, split into discovery and validation cohorts. We confirm and extend known associations between cancer risk and germline RDVs in specific gene-sets, including DNA repair (OR = 1.50; *p*-value = 8.30e-07; 95% CI: 1.28–1.77), cancer predisposition (OR = 1.51; *p*-value = 4.58e-08; 95% CI: 1.30–1.75), and somatic cancer drivers (OR = 1.46; *p*-value = 4.04e-06; 95% CI: 1.24–1.72). Furthermore, personal RDV load in these gene-sets associated with increased risk, younger age of onset, increased M1 macrophages in tumor and, increased tumor mutational burden in specific cancers. Our findings can be used towards identifying high-risk individuals, who can then benefit from increased surveillance, earlier screening, and treatments that exploit their tumor characteristics, improving prognosis.

## Introduction

Inherited genetic variants play an important role in cancer susceptibility. These variants are associated with disease risk in a spectrum, from common variants that tend to have weak effects, to rare variants (<0.5% minor allele frequency, MAF) with often large effects^1,2^. In fact, some studies suggest that greater than 95% of variants predicted to be functionally important are rare^3,4^. In addition to their high penetrance, rare variants are also abundant^3^. Studies in cancer prone families were the first to identify rare, deleterious variants (RDVs) with statistically significantly elevated cancer risk. Well-known examples include *BRCA1/2* in inherited breast and ovarian cancer syndrome^5^, DNA mismatch repair genes in Lynch syndrome^6^, *TP53* in Li-Fraumeni syndrome^7^, and *APC* in familial adenomatous polyposis^8^. Genetic screening for these inherited cancer syndromes has constituted one of the first applications of genomics in precision medicine, as it allows tailored cancer screening, prevention, and in certain cases, therapies^5–8^. While the vast majority of genes identified this way and currently screened for in the cancer clinic are in DNA damage repair (DDR) genes, RDVs in genes for which cancer risks are less well-characterized present a challenge in the clinic^9^.

While genome-wide association studies (GWAS) have identified multiple susceptibility loci as determinants of increased cancer risk in relatively large cohorts, these studies examine associations with common variants only, which typically have modest effects that explain only a small fraction of heritability. RDVs can have larger effect sizes than common variants, yet large cohorts are needed to obtain statistical power for conclusive analyses on their cancer risk, which are often prohibitive. More recently, next generation sequencing studies by large consortia have produced and aggregated data from thousands of germlines and matched tumors. These studies have revealed many germline risk variants^10–14^, and provide a rich resource for investigating the association of RDVs with cancer risk^11,12,15,16^. Our team has previously utilized such resources in focused studies on lung cancer, which revealed that RDVs in *ATM* increase risk for lung adenocarcinomas^16^ and in *Fanconi Anemia (FA)* genes for lung squamous cell carcinomas^15^. In these studies, we addressed the issue of low power observed in single, recurrent RDV studies by conducting case-control analyses for RDVs that may affect risk of cancer cumulatively as part of a gene or gene group. We reason that a similar approach focused on RDVs will provide novel insights into risk across cancers, as it is unlikely to miss weaker associations. To better characterize the roles of RDVs in cancer risk and other cancer-related outcomes, it is paramount to comprehensively assess RDVs in such cancer germline datasets, including analysis of the effects of RDVs aggregated per each individual. This will enable the development of new predictive tools and precision preventive strategies for the clinic.

**Fig. 1:**
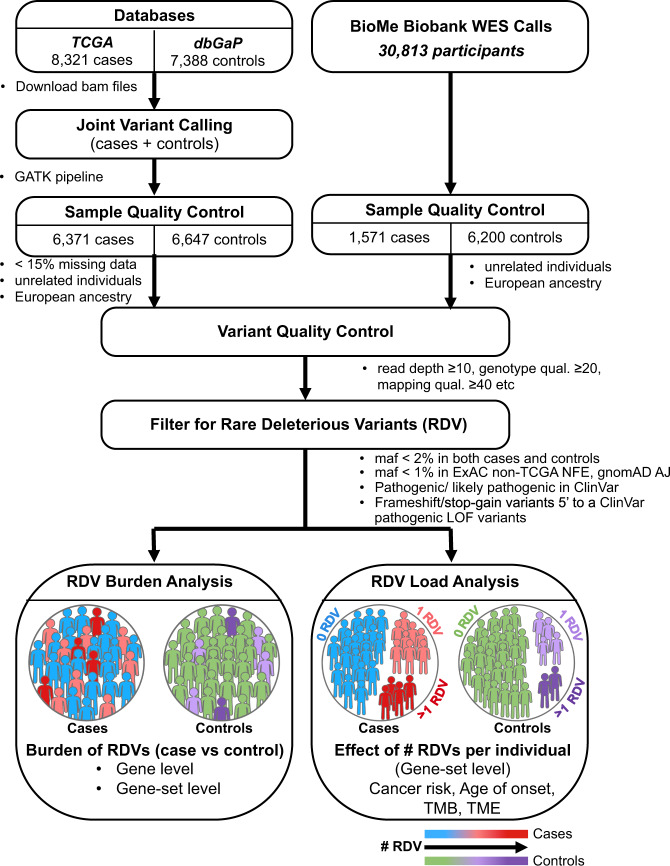
Study design. Flowchart of the study pipeline to identify rare deleterious variants (RDVs) and to perform RDV burden and RDV load analyses.

Towards this end, we have performed the largest-to-date germline sequencing data analysis in a cancer case-control setting, aggregating existing whole-exome sequencing (WES) datasets on 20,789 participants, split into discovery and validation cohorts. In discovery analysis, we processed germline WES datasets on 15,709 participants (13,018 total post-Quality Control, with 6371 cases and 6647 controls) where the cases span 24 different cancers from The Cancer Genome Atlas, TCGA^17^. We then replicated our discovery cohort findings in an independent validation cohort, by using WES data from 7771 participants (1571 cases, 6200 controls) from the Icahn School of Medicine at Mount Sinai (ISMMS) BioMe Biobank. In these studies, as the low frequencies of RDVs make genome-wide discovery difficult without large cohorts, we investigated their roles by collapsing RDVs at gene and gene-set levels for cohort-level *RDV burden* (RDVs in cases vs. controls). Furthermore, unlike previous studies that compared RDVs in cases only to population-level databases for controls, our variant calling in a case-control setting enabled us to identify the number of RDVs, or *RDV load* for each control as well as case participant, and thereby to study the association of personal RDV load with cancer risk (>1 RDV vs. 1 RDV vs. 0 RDV). In Fig. 1, we graphically represent the RDV burden and RDV load concepts. Finally, we studied the association of personal RDV load in cancer individuals with their age of disease onset, tumor mutation burden and cellular composition of tumor immune microenvironment.

RDV burden analysis results revealed that individuals with RDVs in specific cancer genes and gene-sets are at increased risk for cancers. While TCGA germline RDVs have been categorized previously^12^, this study reports result where germline variants are processed in a case-control setting, which enabled us to study associations between personal RDV load and cancer risk. Importantly, this study also replicates germline discovery case-control cohort findings in an independent case-control validation cohort.

Overall, our multi-scale analysis results first confirm known associations in TCGA^12^ and then provide novel observations on the associations of germline RDVs in specific gene-sets with cancer risk. Furthermore, we show that having a germline RDV load in certain gene-sets (i.e. RDVs in more than one gene in the same pathway) is a potential biomarker for younger age of disease onset, tumor mutational burden (TMB), and characteristics of the tumor immune microenvironment (TME) in a dose-dependent manner (i.e. >1 RDV vs. 1 RDV vs. 0 RDV).

## Results

### Germline variant calling in a case-control setting identified sites of rare, deleterious variants (RDVs) and their genotypes across the cohort

**Fig. 2:**
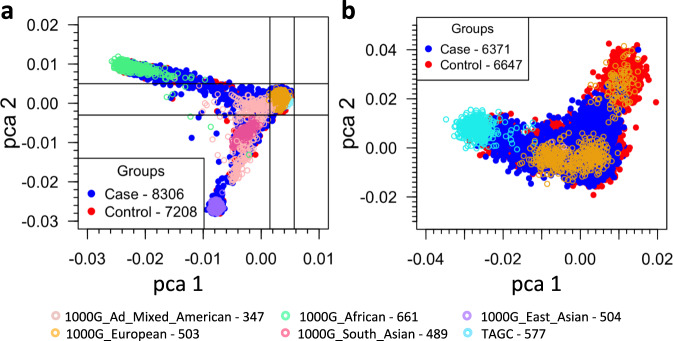
Principal component analyses (PCA) of the study cohort and the gated study cohort. PCA based on common SNPs (MAF ≥ 0.05) showing the top two principal components of (**a**) the study cohort together with 1000 Genomes and The Ashkenazi Genome Consortium (TAGC) samples and of (**b**) the gated samples from the study cohort with European ancestry (6371 cases and 6647 controls).

In the discovery cohort, to enable case-control analyses (Fig. 1) while avoiding biases potentially introduced by different calling algorithms, we first realigned and called variants in the germline WES data in a case-control setting. These included 8321 TCGA cases (Supplementary Table 1), and 7388 controls from dbGaP (Supplementary Table 2), for a total of 15,709 participants. To reduce confounding due to population stratification, we focused on those participants in the discovery cohort who comprised the largest group by ancestry, which clustered via principal component analysis (PCA) with individuals of known European ancestry (Fig. 2). After sample and variant QC (see methods), we observed 941,609 variants (Supplementary Table 3) in 17,507 genes across the autosomes and X chromosomes of 13,018 participants (6371 cases and 6647 controls) (clinical characteristics in Table 1). We note that recently an independent multi-cancer analysis of TCGA cases^12^ (‘case-only analysis’) called each case sample separately (rather than calling variants across all samples in a case-control setting) and used the union of several calling software packages for variant identification. Unlike that case-only approach, our calling approach in a case-control setting can distinguish between instances where a variant site is wild type and where it does not have enough sequence coverage to make a call.

**Table 1:** Characteristics of samples in the case-control study cohorts.

Variables		TCGA-dbGaP cohort		BioMe cohort	
		Cases (6371)	Controls (6647)	Cases (1571)	Controls (6200)
Gender	Male	3099 (48.64%)	4034 (60.69%)	690 (43.92%)	3150 (50.81%)
	Female	3230 (50.70%)	2610 (39.27%)	881 (56.08%)	3050 (49.19%)
	Missing	42 (0.66%)	3 (0.05%)	0	0
Age	Mean (yrs)	60.20	57.54	–	–
	Unknown	86 (1.35%)	4509 (67.84%)		
Smoking	Never	448 (7.03%)	322 (4.84%)	1391 (88.54%)	5418 (87.39%)
	Yes	1491 (23.40%)	746 (11.22%)	174 (11.08%)	744 (12.00%)
	Unknown	4432 (69.57%)	5579 (83.93%)	6 (0.38%)	38 (0.61%)

To compare these two approaches in more detail, we focused on data from 6275 TCGA participants that were analyzed by both methods. From our case-control analysis, after QC we identified 588,287 variants for which at least one alternate allele was called. Of these, 460,373 (78%) were also called by the case-only analysis, while 22% were unique to our calling. Conversely, the case-only analysis call set, which used several genotype callers and took the union of calls, identified 1,681,769 variants, of which 27% were also called by our approach (Supplementary Fig. 1). To estimate the fraction of variants unique to each of these call sets that represent true variant sites versus spurious false positives, we used the imputation panel from the TOPMed consortium^18^ as an external reference. This panel was generated from a large set of participants of diverse ancestry for whom whole genome sequencing (WGS) data are available. Importantly, TCGA participants were not included in this panel. As this panel has been shown to enable imputation of variants at low MAF (0.01%)^19^, we reasoned that it would allow us to assess the quality of many of the low-MAF calls in the call sets. Of the 460,373 germline variants that were called in both the case-control and the case-only analyses, 67% were on the TOPMed panel (Supplementary Table 4). Most importantly, at the variant level, a greater fraction of the alternate alleles at sites found uniquely in our case-control data were concordant with TOPMed compared to the case-only approach (Supplementary Table 5).

Based on the comparison results between the two calling approaches, we proceeded with our case-control analysis approach to identify germline RDVs that associate with cancer risk relative to controls. Overall, we identified 7241 RDVs (MAF < 2% in cases or controls, MAF ≤ 1% in ExAC non-TCGA Non-Finnish European population and MAF ≤ 1% gnomAD Ashkenazi Jewish population) across 1787 genes in the discovery cohort. Similarly, focusing on 1571 cases and 6200 control participants of European ancestry in the validation cohort, we identified 5766 RDVs in 1814 genes.

### Gene-set level burden analysis revealed that RDVs in cancer predisposition, DNA damage repair, *Fanconi Anemia* and somatic cancer driver gene-sets are associated with cancer risk

**Fig. 3:**
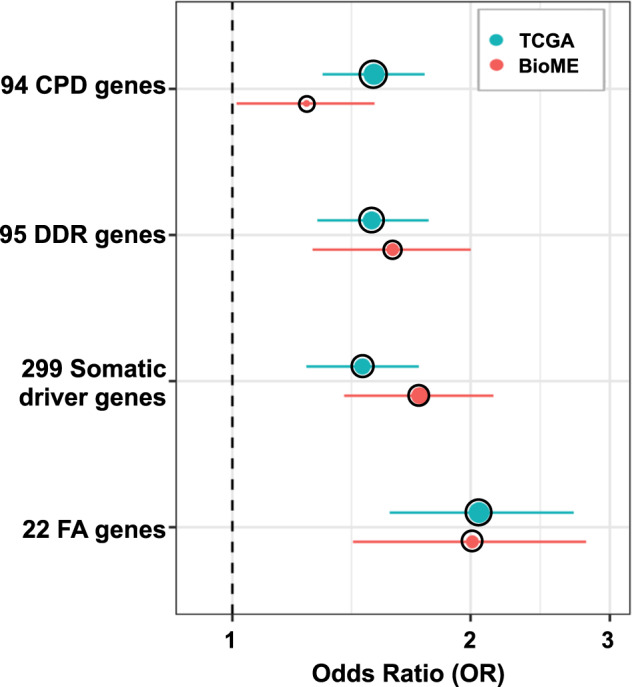
Gene-set level rare, deleterious variant (RDV) burden in the discovery (blue) and validation (red) cohorts. The whiskers span the 95% confidence interval for OR values (penalized logistic regression). The black circle outline indicates significant burden *p* ≤ 0.05.

Though we performed tests at the levels of individual genes, we did not find any significant (*p* ≤ 0.05), replicable associations beyond the well-known association of *BRCA1* (discovery cohort: OR = 2.91; *p*-value = 1.14e-04; 95% CI: 1.67–5.32, validation cohort: OR = 2.91; *p*-value = 1.06e-03; 95% CI: 1.55–5.35) and *BRCA2* (discovery cohort: OR = 3.04; *p*-value = 9.35e-04; 95% CI: 1.55–6.46, validation cohort: OR = 2.50; *p*-value = 1.04e-03; 95% CI: 1.46–4.18) with breast and ovarian cancer risk (Supplementary Data 1). Instead, we hypothesized that collapsing RDVs at the gene-set level would be more powerful and enable better understanding of risk-associated biological processes. Therefore, we compared the RDV burden (Table 2, Fig. 3) in a priori defined gene groups (Supplementary Table 6). We first tested the set of 94 genes in the TruSight Cancer Gene panel (Illumina https://www.illumina.com/products/by-type/clinical-research-products/trusight-cancer.html), which is often used in genetic testing clinics, and observed statistically significant RDV burden (*p* ≤ 0.05) across cancers in the discovery cohort (OR = 1.51; *p*-value = 4.58e-08; 95% CI: 1.30–1.75), which replicated in the validation cohort (OR = 1.24; *p*-value = 0.04; 95% CI: 1.01–1.51). Similarly, when we focused on 95 DNA repair genes involved in known functional DDR pathways^20^, which included those known to associate with autosomal dominant cancer predisposition (CPD) syndromes (20 genes)^21^ (Supplementary Table 6), we observed a statistically significant enrichment of RDVs in cases compared to controls in both discovery (OR = 1.50; *p*-value = 8.30e-07; 95% CI: 1.28–1.77) and validation cohorts (OR = 1.59; *p*-value = 1.17e-04; 95% CI: 1.26–2.00). Next, we hypothesized that germline mutations in genes with known somatic mutations may increase cancer risk, and tested the germline RDV burden on 299 known somatic cancer driver (SCD) genes^22^. We again observed a statistically significant burden of RDVs across cancers in cases vs. controls in the discovery cohort (OR = 1.46; *p*-value = 4.04e-06; 95% CI: 1.24–1.72), which replicated in the validation cohort (OR = 1.72; *p*-value = 2.00e-06; 95% CI: 1.38–2.14).

**Table 2:** Gene-set level rare, deleterious variant (RDV) burden in the study cohorts.

	TCGA-dbGaP Cohort		BioMe Cohort	
	Cases (6371)	Controls (6647)	Cases (1571)	Controls (6200)
	**94 Cancer predisposition genes**			
# Variants	274	186	88	192
# Genes	57	48	35	51
# Unique individuals	464 (7.28%)	326 (4.90%)	136 (8.66%)	439 (7.08%)
OR (*p*-value) [95% CI]	1.51 (4.58e-08) [1.30–1.75]		1.24 (0.04) [1.01–1.51]	
	**95 DNA damage repair genes**			
# Variants	254	181	82	160
# Genes	41	36	29	35
# Unique individuals	374 (5.87%)	269 (4.05%)	108 (6.87%)	273 (4.40%)
OR (*p*-value) [95% CI]	1.50 (8.30e-07) [1.28–1.77]		1.59 (1.17e-04) [1.26–2.00]	
	**299 Somatic cancer driver genes**			
# Variants	231	154	88	169
# Genes	59	52	40	59
# Unique individuals	377 (5.92%)	272 (4.09%)	125 (7.96%)	294 (4.74%)
OR (*p*-value) [95% CI]	1.46 (4.04e-06) [1.24–1.72]		1.72 (2.00e-06) [1.38–2.14]	
	**22 Fanconi Anemia genes**			
# Variants	115	58	32	69
# Genes	15	13	8	13
# Unique individuals	163 (2.56%)	85 (1.28%)	52 (3.31%)	103 (1.66%)
OR (*p*-value) [95% CI]	2.05 (6.14e-08) [1.58–2.70]		2.01 (1.07e-04) [1.42–2.80]

Based on our earlier findings^15^ that RDVs in *Fanconi Anemia (FA)* genes increased risk for squamous lung cancer (LUSC), we also investigated the burden of *FA* (subset of 22 DDR genes) RDVs in other tissues and multi-cancer. Consistent with our LUSC findings, we observed an increased RDV burden cross-cancers in cases vs. controls in both discovery (OR = 2.05; *p*-value = 6.14e-08; 95% CI: 1.58–2.70) and validation (OR = 2.01; *p*-value = 1.07e-04; 95% CI: 1.42–2.80) cohorts. In the discovery cohort, this signal was significantly driven by cancers of the breast, bladder, stomach and ovary (Supplementary Data 2). Furthermore, the 9 *FA* core complex genes and 11 *FA* genes involved in DNA repair both had a statistically significant signal cross-cancers in the discovery cohort (OR = 1.93; *p*-value = 0.02; 95% CI: 1.10–3.49 and OR = 2.12; *p*-value = 2.15e-06; 95% CI: 1.54–2.93, respectively). The finding on 11 *FA* genes involved in DNA repair further replicated in the validation cohort (OR = 2.48; *p*-value = 3.14e-06; 95% CI: 1.71–3.56), while on 9 *FA* core complex genes trended in the expected direction (OR = 1.18; *p*-value = 0.74; 95% CI: 0.41–2.88). Next, to make sure the signals we observed in these gene-sets (CPD, DDR, SCD and *FA*) were not solely driven by *BRCA1/2*, as a sensitivity analysis we removed *BRCA1/2* and repeated the burden analyses, and observed that these gene-sets were still significant (Supplemental Table 7, Supplementary Fig. 2). Furthermore, our analysis of the MSigDB gene-sets (below) supported our findings on the association of the *FA* gene-set with cancer risk. Note that while we further tested for the confounding effects of gender for all gene-sets multi-cancer, we did not observe a noticeable difference in the OR results for any gene-set (Supplementary Fig. 3). We provide the gender distribution in Supplementary Table 8.

Finally, we performed a similar gene-set level burden analysis (Supplementary Data 3) for sets of cancers grouped by histological or anatomical relation, including pan-gastrointestinal^23^, pan-kidney^24^, pan-gynecological^25^, and pan-squamous^26^. Pan-gastrointestinal and pan-gynecological cohorts were associated with significant (*p* ≤ 0.05) cancer risk with RDVs in all four gene-sets (CPD, DDR, SCD and *FA*). Furthermore, in pan-kidney and pan-squamous analyses, we observed significant burden of RDVs in cases compared to controls in CPD and *FA* gene-sets, respectively (Supplementary Data 3).

### Data-driven analysis of 17,810 gene-sets in MSigDB again identified RDV *burden* in *Fanconi Anemia* genes associated with increased cancer risk

Next, to ensure we did not miss any additional gene-sets with significant RDV burden in cases vs. controls, we performed a data-driven exome-wide gene-set analysis. For this purpose, we tested all gene-sets (17,810) contained in the Molecular Signatures Database (MsigDB)^27^ (Supplementary Data 4). Consistent with our observations on pre-selected gene-sets, the strongest signal was in *Fanconi Anemia* pathway genes from REACTOME (*p* = 1.4e-11; OR = 2.54; 95% CI = 1.92–3.40) in the discovery cohort, which was further replicated in the validation cohort (*p* = 1.2e-05; OR = 2.21; 95% CI = 1.56–3.09). Other cancer-related pathways, for the majority including the strongest DDR genes, were found statistically significant after adjusting for multiple comparisons. Of note in the discovery cohort, we observed significant risk association for RDV burden in the set of genes targeted by the eukaryotic translation initiation factors *EIF4EBP1* and *EIF4EBP2* (OR = 1.43; *p*-value = 4.4e-05; 95% CI: 1.20–1.69). While we did not observe significant association in the validation cohort, we still observed higher frequency with the same direction of effect in cases with RDVs compared to controls (OR = 1.14; *p*-value = 0.39; 95% CI: 0.85–1.51).

### Germline RDV *load* in key cancer genes is a potential marker for increased cancer risk

**Fig. 4:**
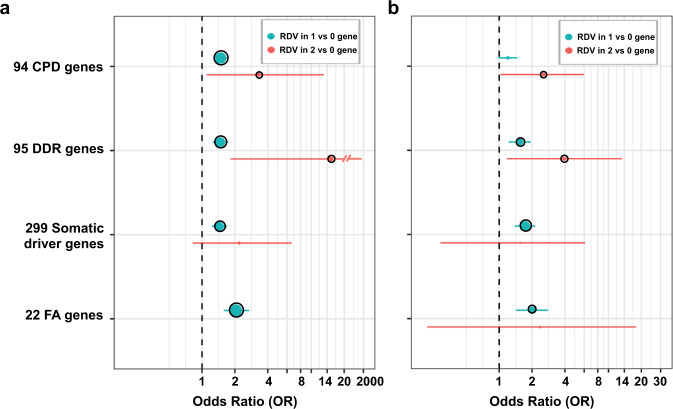
Cancer risk based on RDV load. **a** Discovery cohort; **b** Validation cohort. The whiskers span the 95% confidence interval for OR values (penalized logistic regression). The black circle outline indicates significant burden *p* ≤ 0.05.

RDVs in cancer are usually considered in a binary context: a patient either has an RDV in a gene of interest or does not (similar to our RDV burden analysis). Given that even when the penetrance of RDVs in cancer risk is high, it is not absolute (i.e., some individuals with RDVs in known cancer predisposition genes will never develop cancer), we hypothesized that the accumulation of RDVs within a set of related genes in an individual could increase their cancer risk. We refer to this concept as personal “germline RDV load”. We hypothesized that an increased personal germline RDV load in particular gene-sets is associated with an increased risk of cancer. The premise of this hypothesis is that the RDVs damage, but do not fully destroy a particular pathway; therefore, additional RDVs in the same pathway add to the damage and increase cancer risk further. To test this hypothesis, we evaluated the associations between the participant-level RDV load *within* gene-sets (i.e., CPD, DDR, SCD and *FA)* (Supplementary Table 6) and cancer risk (Fig. 4 and Supplementary Table 9). For each gene-set, we divided the participants into three participant groups: (i) participants with no RDVs; (ii) participants where RDVs occurred in only one gene; and (iii) participants with RDVs in two or more genes (Supplementary Table 9), recognizing that the small number of participants with RDVs in >1 gene makes precise estimation of the effect of RDV load difficult. Consistent with our hypothesis on RDV load, for each studied gene-set, we observed a greater association with cancer risk for participants that had higher number of genes with germline RDVs, as shown in Fig. 4 and Supplementary Table 9 (except for *FA* due to the limited number of genes in this gene-set). Notably, all our RDV load findings replicated in the validation cohort. Note that in SCD and *FA* gene-sets, we did not observe significant cancer risk association (*p*-value ≤ 0.05) with individuals with RDVs in more than one gene.

**Fig. 5:**
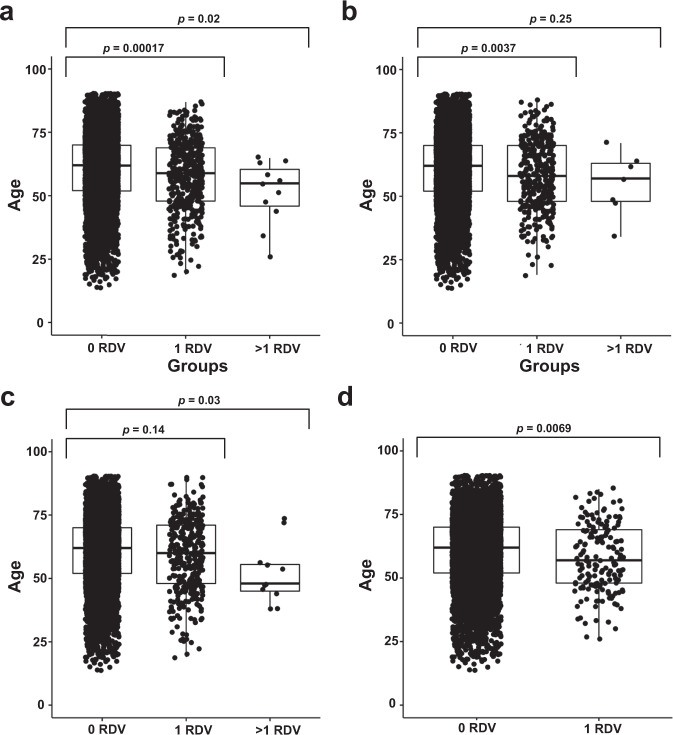
Comparison of age of diagnosis based on germline RDV load in TCGA cases. **a** Cancer predisposition genes **b** DNA damage repair genes **c** Somatic cancer driver genes **d** Fanconi Anemia genes. *p*-values are calculated based on Mann–Whitney *U*-test. Boxplot elements: center line indicates median; box limits represent lower (25th percentile) and upper (75th percentile) quartiles; whiskers extend to 1.5 times the interquartile range.

We further hypothesized that a higher RDV load in these gene-sets is associated with a younger age of diagnosis. To test this hypothesis, we used the detailed clinical information available on the discovery cohort of TCGA participants. We evaluated the associations between each participant’s germline RDV load with their age at cancer diagnosis, and observed that those participants with a germline RDV indeed exhibited a statistically significant early age of diagnosis than those individuals without RDVs (Fig. 5) in CPD, DDR and *FA* gene-sets. Additionally, we observed that the individuals with high RDV load (>1 RDV) in CPD and SCD gene-sets had significantly younger age of diagnosis than those individuals without RDV.

### Germline RDV load is associated with altered tumor immune microenvironment and increased tumor mutation burden

**Fig. 6:**
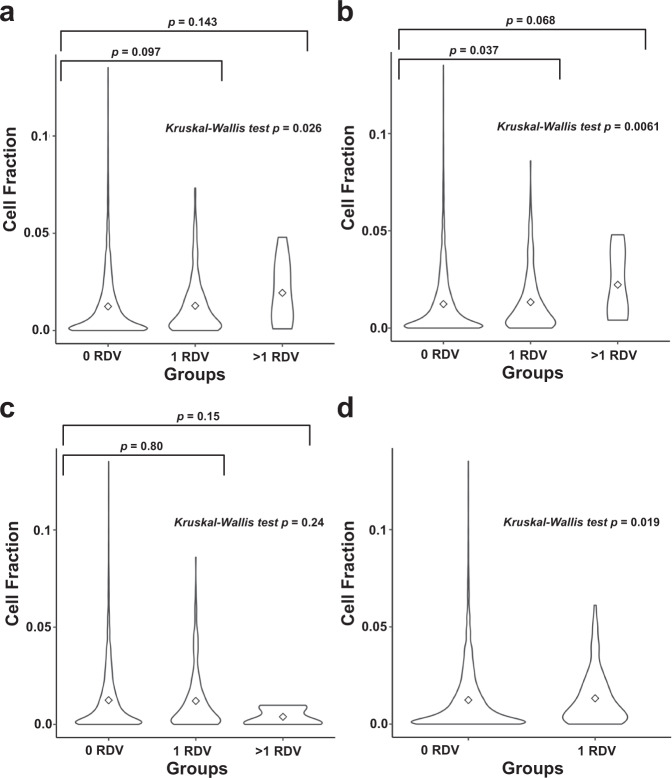
Comparison of M1 macrophages cell fraction in tumor based on germline RDV load in TCGA cases. **a** Cancer predisposition genes **b** DNA damage repair genes **c** Somatic cancer driver genes **d** Fanconi Anemia genes. The marker in the violin plot indicates mean.

Next, in light of recent findings on the impact of germline variants on tumor immune microenvironment (TME) in various cancers^28–30^, we tested the association of increased germline RDV load in CPD, DDR, SCD and *FA* gene-sets with TME immune cell fractions. Briefly, to avoid any potential biases from B-cell immune signatures in blood cancers, we focused on 6277 participants with solid tumors in TCGA by excluding 94 TCGA participants with hematological malignancies. We then utilized previously reported annotations on the 22 infiltrating immune cell types on solid tumors of TCGA participants^31^, based on analysis by the CIBERSORT tool that used their tumor RNA-sequencing data^32^. Next, for each participant, we tested the association of tumor immune cell fraction with their germline RDV load in the CPD, DDR, SCD and *FA* gene-sets (Supplementary Fig. 4, Supplementary Data 5). Remarkably, the germline RDV load in CPD, DDR or *FA* genes exhibited the strongest association with increased levels of M1 macrophages in the TME. Specifically, we observed that participants with a germline RDV in DDR or *FA* gene-sets developed tumors with a statistically significantly higher fraction of M1 macrophages (Fig. 6), compared to participants with no RDV. Additionally, we observed that the individuals with high RDV load (>1 RDV) in CPD and DDR gene-sets had higher fraction of M1 macrophages than those individuals without RDV although it was not statistically significant. This signal was mostly driven by the increased levels of the chemokine ligands *CXCL10/11*. We provide the complete set of results in Supplementary Data 6. We next asked whether increased M1 macrophages in TME of participants with solid tumors associated with survival (Supplementary Table 10). We observed that those participants with increased M1 macrophages exhibited worse survival (*p* ≤ 0.05 without correction for multiple testing) in brain lower grade glioma, kidney renal clear cell carcinoma and kidney renal papillary cell carcinoma.

Finally, since tumor mutation burden (TMB) is currently being investigated as a biomarker for durable response to life-extending cancer immunotherapies^33^, we also tested the association of germline RDV load in CPD, DDR, SCD and *FA* gene-sets with TMB for each cancer type with solid tumors (Supplementary Data 7). We observed the most significant TMB association with germline RDV load in *FA* genes in breast cancer patients (*p* = 0.00052). Overall, germline RDVs associated with TMB in: (i) DDR, CPD and SCD genes in colon cancer; (ii) DDR genes in kidney clear cell and cervical cancers; (iii) CPD genes in kidney papillary cell cancer; (iv) SCD genes in stomach and ovarian cancers; and (v) *FA* genes in breast, cervical, ovarian and stomach cancers. This signal for *FA* genes was mostly driven by *BRCA1/2*; when we removed *BRCA1/2* and reperformed the analysis, we still observed associations of germline RDV load in *FA* genes and TMB for stomach cancer. Details are in Supplementary Data 7.

## Discussion

Here, we have performed the largest-to-date case-control WES study to identify germline rare, deleterious variants (RDVs) in specific genes and gene-sets that associate with multi-cancer risk and to further investigate whether this risk increases with the number of RDVs an individual has. The study findings were made possible by performing variant calling analysis in a case-control setting by including thousands of controls, which allowed the examination of combinations of variants for individual control participants. We then used an independent pan-cancer cohort from the ISMMS BioMe Biobank for validation. To examine the cumulative effects of RDVs in functionally related gene groups, we used a gene-set based variant collapsing approach. Specifically, we focused both on a few cancer-associated gene groups, as well as in a function-agnostic manner on all 17,810 gene-sets in MSigDB^27^. For cancer-associated gene groups, mindful of the fact that many risk variants have been shown to exhibit tissue specificity^34^, we tested RDVs in cancer predisposition (CPD) and DNA damage repair (DDR) genes, which we hypothesized would pleiotropically associate with risk across cancer sites, due to the accumulation of mutations that fail to be properly repaired^35^. In addition, we tested whether germline RDVs in genes with known somatic mutations (somatic cancer drivers, SCDs) increase cancer risk^22^. Finally, based on our earlier findings^15^ that RDVs in *Fanconi Anemia (FA)* genes are associated with lung squamous cancer risk, we tested RDVs in the *FA* genes. Remarkably, we observed increased cancer risk associated with RDVs in all four tested gene-sets (CPD, DDR, SCD, and *FA*) in both discovery and validation cohorts. Furthermore, our analysis of the MSigDB gene-sets supported our findings on the association of the *FA* gene-set with cancer risk.

Our results demonstrate the value of variant calling in a case-control setting in germline risk variant discovery. Notably, unlike prior works^12^ that have simply compared germline variant frequencies in TCGA cancer individuals to those in databases such as ExAC^36^ non-TCGA or gnomAD^37^ as controls, we performed germline variant calling in a case-control setting. Doing so enabled us to identify control participants with more than one RDV in a given gene or gene-set and to examine the role of personal *RDV load* in cancer risk (see Fig. 1). This is a significant finding, which prior works^12^ were unable to examine. While multiple studies have focused on the somatic tumor mutation burden (TMB), our study highlights the critical importance of germline RDV load in key cancer gene-sets. The association of increased RDV load in CPD, DDR, *FA* and SCD gene-sets with increased personalized cancer risk in both discovery and validation cohorts have important implications for our understanding of how germline genetic factors govern cancer risk, and can impact the clinical management of cancer patients with one or more germline RDVs, and their families.

We further observed that personal RDV load in specific gene-sets associated with age of disease onset, tumor immune microenvironment (TME) and TMB in TCGA matching tumor data. That individuals who have RDVs in CPD, DDR or *FA* genes have statistically significantly earlier age of diagnosis than those without warrants research in whether these individuals should start screening and surveillance efforts at younger ages than the currently recommended guidelines for the general public.

TME and TMB are two tumor characteristics important in immunotherapy response and prognosis. Recent studies show that germline variants can shape certain immune features within the TME of solid tumors^28–30^. Consistent with and complementary to these observations, our results show that the personal germline RDV load of a cancer patient is associated with their TME (see Supplementary Fig. 4, Supplementary Data 5). Interestingly, the germline RDV load in CPD, DDR or *FA* genes exhibited the strongest association with increased levels of M1 macrophages in the TME (Fig. 6). Unlike T-cells which may or may not be in the TME^38^, macrophages are often present in tumors as the most dominant cells, including tissue resident cells and infiltrating cells, with relative proportions varying in different patients. However, the potential impact of TME macrophage elevation is currently somewhat controversial, as the historical dichotomy between good (M1) vs. bad (M2) subsets is being challenged by more granular data on macrophage subsets and lineage based on single-cell transcriptomics^39^. While increased levels of M2 macrophages in tumors generally associate with poor survival^40,41^, the levels of M1 macrophages have so far not been associated with poor outcome. M1 macrophages have been shown to likely contribute to a more immunogenic tumor environment by providing activating signals, including Th1 polarizing cytokines or chemokines^42^. They also are likely to active via their phagocytic function, either directly through eating up tumor cells and contributing to antigen presentation and priming of T cells as well, or via antibody dependent cell-mediated cytotoxicity^43^. As TME can impact response to standard-of-care therapies^44^, including chemotherapy, radiation and angiogenic inhibitors, and since clinical trials that examine the combinations of these treatments are currently on-going, our results warrant further investigations into identifying specific macrophage markers that may correlate with immunotherapy treatment efficacy and prognosis of individuals with germline RDV load in CPD, DDR or *FA* genes in solid tumors.

We further observed statistically significant association of RDV load with TMB in different gene-sets for different cancer types (see Supplementary Data 7). Since TMB has been suggested as a biomarker for durable response to cancer immunotherapies^33,45^, these results further support our findings on the potential importance of RDV load in specific gene-sets in shaping tumor immune characteristics (Supplementary Data 5). For example, in colon cancer RDV load in most gene-sets we tested associated with increased TMB.

Finally, we asked whether RDV load is associated with survival. While we had limited statistical power to answer this question directly, we did observe that increased M1 macrophages (which correlated with RDV load) associated with worse survival (*p* ≤ 0.05 without correction for multiple testing, Supplementary Table 10) in specific cancers (brain lower grade glioma, kidney renal clear cell carcinoma and kidney renal papillary cell carcinoma). The functionality of macrophages in these histologies compared to other tumor types in relation to antigen presentation capacity or inflammatory potential remains to be further explored. Future studies in larger, independent cohorts are needed for validation of this finding and will help better understand the nature of interactions between germline RDV load, tumor characteristics and survival.

This study should be considered in the context of its limitations. First, the multi-cancer analysis needs to consider the overrepresentation of rarer cancers in the TCGA data. Thus, strong association signals specific to these less common cancers in the TCGA data may be weaker when studied in a cohort reflective of the population incidences of cancers of various sites. Similarly, inclusion of some cancer types with small sample size increased the heterogeneity of our study, risking a diluted signal for associations with more common cancers. Second, while focusing on variants annotated as pathogenic in ClinVar^46^ was necessary to ensure the clinical reliability of our results, it also restricts our analysis to those genes previously known to have a clinical impact. There are variants, such as rs11571833 (p.Lys3326Ter) in *BRCA2*, for which research studies strongly support a role in cancer risk^15,47^ but not annotated as pathogenic in ClinVar. Alternative approaches to identifying pathogenic variants will be needed to address these issues. Third, while we had well-annotated gender data available to study its potential confounding effects, information on other potential confounders such as age and smoking was not available for all controls in the dbGaP studies. Thus, we were unable to investigate their potential confounding effects and cannot distinguish direct genetic effects on cancer risk versus genetic effects on risk factors for cancer. Fourth, our results do not explore the interplay between specific RDVs, RDV load, and environmental and clinical exposures. Future efforts that link genetic information with epidemiological exposure and clinical information from electronic health records (EHR) (e.g. blood measurements; smoking and alcohol history; viral infections, etc.) will be needed to understand such interactions. This work also did not consider the role of common genetic polymorphisms in cancer risk, such as that captured by polygenic risk scores^48,49^. Future efforts towards understanding if and how the penetrance of RDV load (and visa versa) will be impacted with polygenic background could be quite informative^50,51^. We did not include cancer types for which data are currently available in TCGA, but were not available when we first began this project. Finally, the statistical power of our validation was somewhat limited. As data from larger population- or hospital-based BioBanks (e.g. UKBiobank) become available, larger studies to interrogate the role of RDV load in multi-cancer and tissue-specific cancer risk will become possible.

## Methods

### Data sources

For discovery, we used case data from TCGA (Supplementary Table 1) and control data from twelve population-based studies (Supplementary Table 2) in the database of Genotypes and Phenotypes (dbGaP) (http://www.ncbi.nlm.nih.gov/gap, RRID:SCR_002709). Briefly, we downloaded TCGA germline WES bam files from National Cancer Institute Cancer Genomics Hub (cgHub), a predecessor to the Genomic Data Commons which is no longer online. We extracted control fastq files from the NCBI Short Read Archive (SRA) for dbGaP studies listed in Supplementary Table 2. For replication, we used the exome calls from BioMe Biobank^52^ of Icahn School of Medicine at Mount Sinai (ISMMS). The relevant data access committees at NIH under project #8668 approved TCGA and dbGaP data usage. The usage of BioME data in our study (HS# 19-01088) was approved by the Program for the Protection of Human Subjects (PPHS) office at ISMMS as exempt human research (IRB-19-02546).

### Study cohorts

For the discovery cohort, we realigned and called germline variants in a case-control cohort setting for 8321 TCGA cases and 7388 dbGaP controls. Cases included participants with 24 different cancer types of different sample sizes, as listed in Supplementary Table 1. For the control samples, when the underlying dbGaP study was a case-control study focused on a disease, we only included control individuals who did not have that disease in question.

For the validation cohort, we utilized available datasets from ISMMS BioMe^52^, which is an electronic health record (EHR) linked biobank. BioMe encompasses a wide array of phenotypic and genetic data from the diverse population of ISMMS patients with a vast spectrum of medical disorders. WES data (Illumina v4 HiSeq 2500) already exist for 30,813 BioMe participants^52^. Focusing on BioMe participants with existing WES data, we identified cancer patients and healthy controls based on their International Classification of Diseases (ICD)-9 and ICD10 codes^53^, which led to 1,571 cases and 6,200 controls of European ancestry (filtering details below). The clinical characteristics of the cohorts are listed in Table 1. The study design is provided in Fig. 1.

### Discovery cohort variant discovery

We first realigned and called germline variants from WES datasets in a case-control setting using GVCF-based best practices for the genome analysis toolkit (GATK, https://www.broadinstitute.org/gatk/, RRID:SCR_001876) as implemented in a custom pipeline at the Icahn School of Medicine at Mount Sinai (ISMMS)^54^ and previously^15,55,56^. Briefly, we independently aligned all samples to human genome build GRCh37 with BWA^57^ (RRID:SCR_010910), performed indel realignment, duplicate marking and base quality score recalibration using GATK and Picard (RRID:SCR_006525), and finally called to a GVCF file with HaplotypeCaller. The case-control germline variant calling step consisted of calling variants from GVCF files and variant quality score calibration with GATK, where we only included samples for which over 75% of the exome was callable (depth ≥ 20, mapping quality ≥ 10, base quality ≥ 20) and for which there was no evidence of contamination (VerifyBamID < 3%).

### Discovery cohort sample QC

We first removed samples with 15% or more missing genotype data. To filter samples that are duplicates or from first or second degree relative pairs, we performed relatedness analysis with KING software^58^ (RRID:SCR_009251) and then removed a sample from each such pair that had the highest fraction of missing data. To remove any bias that may arise due to systematic ancestry-based variations in allele frequency differences between cases and controls (i.e. population stratification) we used Principal Component Analysis (PCA). Briefly, we first removed indels and rare variants (defined by <5% of minor allele frequency, MAF), using 1000 Genomes dataset^59^ (RRID:SCR_008801) and The Ashkenazi Genome Consortium (TAGC, https://ashkenazigenome.org) as reference. For the remaining variants, we performed linkage disequilibrium (LD) pruning, filtered for a call rate of at least 0.99, and performed PCA with smartpca using EIGENSOFT 5.0.1 software (RRID:SCR_004965). We filtered for the least ancestry-based variation by focusing our downstream analyses on the largest set of case-control individuals clustered within the PCA plot by examining the PCA plot and selecting thresholds on PC1 and PC2 to corresponded to individuals of European ancestry (EA); analogous to flow cytometry we call this approach “gating”. The PCA plots along with the gated region are shown in Fig. 2. To adjust for population-level differences, we used the first two principal components from PCA of the gated individuals as covariates in the burden analyses. After sample QC, 6371 cases and 6647 controls remained.

### Discovery cohort variant-level QC

For participants that passed PCA gating, we focused on ensuring high-quality genotype/variant calls for analysis. For this purpose, we filtered for variants with: read genotype quality ≥20; read depth ≥10; allelic depth of alternate allele ≥4; sites with: quality score ≥50; quality by depth score ≥2; mapping quality ≥40; read position rank sum > –3; mapping quality rank sum > –10 and variant tranche <99%. For heterozygous genotypes, we filtered for alternative allele ratio between 0.30 and 0.70. To reduce any differences between samples in cases and controls, we kept sites that have differential missing variant fraction ≤ 0.05 between the cases and controls. Finally, we kept sites with ≥88% of data (in both cases and controls).

### Validation cohort participant selection

Next, to replicate the discovery cohort findings within an ancestry-matched validation cohort, we focused on those of EA in BioMe. Briefly, we used the PCA performed on the common variants^60^ and gated for EA individuals based on the first two principal components which captured the majority of the variance, resulting in 10,784 BioMe participants of (Supplementary Fig. 5). Next, we also ensured we used data from unrelated participants up to second degree. Finally, we identified participants with cancer based on their ICD9 and ICD10 codes available within BioMe^52^. Specifically, to avoid any false positives, we filtered for participants who had ICD9/10 codes related to cancer at least twice in their diagnosis files on separate dates. To avoid any conflicts in categorizations, we also removed participants with the following diagnoses (or diagnoses of similar nature): benign neoplasms, neoplasm of uncertain behavior and genetic susceptibility to malignant neoplasm. The complete list of ICD9/10 codes used for classification and elimination of cancer diagnosis are listed in Supplementary Data 8. We considered all other participants who were unaccounted for in the above categories as controls. These led to 1571 cases and 6200 control participants of EA in the BioMe cohort.

### Validation cohort data generation and variant QC

BioMe WES data generation and QC steps have been discussed in detail previously^52^. Briefly, we filtered out sites with missingness >0.02 and biallelic sites with allele balance (<0.3 or >0.8). Additionally, to be consistent with the discovery cohort variant QC, we filtered for variants with: read genotype quality ≥20; read depth ≥10; allelic depth of alternate allele ≥4; sites with: quality score ≥50; quality by depth score ≥2; mapping quality ≥40; read position rank sum > –3; mapping quality rank sum > –10; differential missing variant fraction ≤ 0.05 between the cases and controls and site missingness <12% (in both cases and controls).

### Variant filtering (both cohorts)

After sample and variant QC, we focused on rare, deleterious variants (RDVs) with known pathogenicity. To filter out common polymorphisms, we removed any variant present in both case and control cohorts at MAF > 2% or in Exome Aggregation Consortium (ExAC)^36^ non-TCGA Non-Finnish European population at MAF > 1% or in Genome Aggregation Database (gnomAD)^37^ Ashkenazi Jewish population at MAF > 1%. We considered variants that pass these filters to be rare. We then filtered the remaining variants for functional impact based on those present in the ClinVar database^46^ (RRID:SCR_006169) using the Annovar tool (http://annovar.openbioinformatics.org, RRID:SCR_012821). We considered a variant to be deleterious if: (i) it is listed as pathogenic/likely pathogenic in ClinVar; or (ii) it is a frameshift or stopgain variant located 5′ of a variant described to be a pathogenic LOF variant in ClinVar (nonsense and frameshift). We also performed a sensitivity analysis for our MAF cutoff by removing any variant present in both case and control cohorts at MAF > 1% or in ExAC non-TCGA Non-Finnish European population at MAF > 1% or in gnomAD Ashkenazi Jewish population at MAF > 1% and did not observe a difference in the top genes or gene-set level burden analyses (data not shown).

### Statistical analysis

#### Background variation correction

To test for possible background variation between cases and controls, we calculated the tally of rare autosomal synonymous variants per each study participant. We defined synonymous variants as rare at Exac MAF ≤ 0.005% and cohort MAF ≤ 0.05%. Supplementary Fig. 6 provides the distribution and background variation statistics of genes with rare synonymous variants between the cases and controls in both cohorts. We accounted for differences in background variation by using the number of genes with rare synonymous variants of each individual as a covariate during the burden analyses.

#### Gene level RDV burden analyses

Next, to evaluate the cumulative effects of multiple RDVs in each gene, and thereby increase the statistical power to identify cancer risk genes, we performed gene level RDV burden tests. Briefly, to accommodate for data sparsity, we performed aggregate RDV burden for each gene using penalized logistic regression analysis (PLRA), using the logistf package in R (https://cran.r-project.org/web/packages/logistf/index.html). To adjust for background variation, we used the number of genes with rare synonymous variants as a covariate for each individual in both cohorts. Additionally, in the discovery cohort we used the first two principal components as covariates to adjust for population difference. We deemed genes with *p*-value ≤ 0.05 and odds ratio >1 as statistically significant risk genes. We performed the burden analysis in pan-cancer and tissue-specific cancer types. All statistical tests were two-sided.

#### Gene-set level RDV burden analyses

Next, we evaluated the RDV burden of gene-sets that typically play key roles in cancer risk and progression: (i) known cancer predisposition genes (Illumina, https://www.illumina.com/products/by-type/clinical-research-products/trusight-cancer.html); (ii) DNA damage repair genes^20,21^; (iii) somatic cancer driver genes^22^ and (iv) Fanconi Anemia genes^61^. We performed the burden analysis for (i) tissue type specific cancers; (ii) histologically or anatomically related cancer types, including pan-gastrointestinal^23^, pan-kidney^24^, pan-gynecological^25^, and pan-squamous^26^, and (iii) pan-cancer. We provide a complete list of genes in these gene-sets in Supplementary Table 6. For burden analyses, we used PLRA and considered all participants with at least one RDV in a gene within the considered gene-set. Furthermore, for an unbiased data-driven exome-wide gene-set analysis, we also tested all gene-sets (17,810) in Molecular Signatures Database (MSigDB)^27^ using the same RDV burden approach. We considered statistically significant burden as *p*-value ≤ 0.05.

#### Gender effects

The gender breakdown of participants for each cancer type in TCGA is provided in Supplementary Table 8. To study the effect of gender on burden analyses, we first removed samples with missing gender data (Supplementary Table 8) and then used PLRA with gender as an additional covariate. Resulting ORs and *p*-values of all gene-sets are in Supplementary Fig. 3. Please note that we did include gender-biased cancer subtypes breast, prostate, ovarian, cervical and uterine (endometrial and sarcoma) in the multi-cancer analysis of gender effects.

#### Cancer type grouping

While cancer type is traditionally defined based on tissue of origin and histologic type, recently^62^, histologically or anatomically related cancer types have been studied together, including pan-gastrointestinal^23^, pan-kidney^24^, pan-gynecological^25^, and pan-squamous analyses^26^. In light of these studies, we performed gene-set level burden analyses in these four sub-groups. We included 634 patients with stomach, colon or rectum adenocarcinoma for pan-gastrointestinal; 528 patients with clear cell, papillary or chromophobe renal cell carcinoma for pan-kidney; 1392 patients with ovarian serous cystadenocarcinoma, uterine corpus endometrial carcinoma, cervical squamous cell carcinoma and endocervical adenocarcinoma, uterine carcinosarcoma or breast invasive carcinoma for pan-gynecological; and 1038 patients with lung squamous cell carcinoma, head and neck squamous cell carcinoma, or cervical and bladder cancers with squamous differentiation^26^ for pan-squamous analyses. For all of these groupings, we compared the cases with the full set of controls.

#### Germline RDV load effects

Next, we asked whether the accumulation of personal germline RDVs, or “RDV load” of an individual impacts their personal cancer risk. Towards this end, we first divided the discovery cohort participants based on their RDV loads into three groups: participants with (i) no RDVs; (ii) one RDV; and (iii) more than one RDV. We then tested and compared the association of germline RDV load with age of diagnosis between these groups using the Mann–Whitney U test. Next, we asked whether the germline RDV load is associated with tumor immune microenvironment (TME). For this purpose, we used existing datasets^31^ on the relative fraction of 22 different immune cell types within TME across TCGA cancers, as estimated by the CIBERSORT tool^32^ (RRID:SCR_016955), which included 5917 cases. To obtain the total cell fraction in tissue, we multiplied the relative immune cell fractions with leukocyte fraction^31^. We then compared the immune cell fractions between the groups using the Kruskal-Wallis test. Next, to study the effect of M1 macrophages on survival, we used Cox proportional hazards regression model. Finally, to study the effect of germline RDVs on tumor mutation burden (TMB) between the groups, we used Mann–Whitney U test. To calculate the TMB, we used the publicly available TCGA somatic mutations MAF file (mc3.v0.2.8.PUBLIC.maf.gz) which included 6225 cases. We used the TMB definition as the total number of somatic, missense, nonsense, frameshift/inframe mutations per megabase (Mb) of genome examined, with 38 Mb as an estimate of exome size.

### Reporting summary

Further information on research design is available in the Nature Research Reporting Summary linked to this article.

## Supplementary information

Supplementary Tables and Figures

Supplementary Data 1

Supplementary Data 2

Supplementary Data 3

Supplementary Data 4

Supplementary Data 5

Supplementary Data 6

Supplementary Data 7

Supplementary Data 8

REPORTING SUMMARY

## Data Availability

The results here are in part based upon data generated by the TCGA Research Network (https://www.cancer.gov/tcga) and datasets from dbGaP (http://www.ncbi.nlm.nih.gov/gap) through dbGaP accession numbers phs000209, phs000276, phs000296, phs000298, phs000424, phs000654, phs000687, phs000806, phs000876, phs000971, phs001000, phs001101. BioMe BioBank (http://icahn.mssm.edu/research/ipm/programs/biome-biobank) WES data from Mount Sinai Health system requires PPHS/IRB approval.

## Code availability

All analyses were performed utilizing standard publicly available software. Any specific analysis code details are available from the authors upon request.
