# Supplementary material for: Germline rare deleterious variant load alters cancer risk, age of onset and tumor characteristics
Source: NPJ Precis Oncol. 2023 Jan 27;7:13. doi: 10.1038/s41698-023-00354-3 (PMC9883433; doi:10.1038/s41698-023-00354-3)
Supplement: Supplementary file 1 — Supplementary Tables and Figures [file 41698_2023_354_MOESM1_ESM.pdf]

Germline rare deleterious variant load alters cancer risk, age of onset and tumor characteristics

Myvizhi Esai Selvan[1,2,4], Kenan Onel[1], Sacha Gnjatic[3,4], Robert J. Klein[1] and Zeynep H. Gümüş[1,2,4*]

[1]Department of Genetics and Genomic Sciences, Icahn School of Medicine at Mount Sinai, New York, NY, 10029, USA

[2]Center for Thoracic Oncology, Tisch Cancer Institute, Icahn School of Medicine at Mount Sinai, New York, NY, 10029, USA

[3]Oncological Sciences, Tisch Cancer Institute, Icahn School of Medicine at Mount Sinai, New York, NY, 10029, USA

[4]Precision Immunology Institute, Icahn School of Medicine at Mount Sinai, New York, NY, 10029, USA

List of Supplementary Figures

Supplementary Figure 1. Comparison of the variants identified in 6,275 TCGA cases from our case-control analysis and previous case-only analysis

Supplementary Figure 2. Gene-set level rare, deleterious variant (RDV) burden in the study cohorts with and without BRCA1/2 RDVs a) Discovery cohort b) Validation cohort

Supplementary Figure 3. Effect of gender on gene-set level burden of rare, deleterious variant burden in the study cohorts

Supplementary Figure 4. Comparison of immune cell fractions (from CIBERSORT) based on germline RDV load in TCGA cases for different gene-sets

Supplementary Figure 5. Gating of individuals of European ancestry in the BioMe cohort

Supplementary Figure 6. Tally of genes with per-sample rare synonymous variants between cases and controls

List of Supplementary Tables

Supplementary Table 1. Distribution of samples in TCGA (https://www.cancer.gov/tcga)

Supplementary Table 2. dbGaP (http://www.ncbi.nlm.nih.gov/gap) control cohort

Supplementary Table 3. List of variants identified during germline variant calling in a case-control setting in the discovery cohort after sample and variant QC (6,371 cases and 6,647 controls)

Supplementary Table 4. Count of variants identified in prior case-only approach, our case-control approach and TOPMed

Supplementary Table 5. Comparison of the genotypes identified in prior case-only approach, our case-control approach and TOPMed

Supplementary Table 6. List of genes used in gene-set level burden analyses

Supplementary Table 7. Gene-set level rare, deleterious variant (RDV) burden in the study cohorts without BRCA1/2 RDVs

Supplementary Table 8. Distribution of males and females in the study cohorts

Supplementary Table 9. Gene-set level rare, deleterious variant (RDV) burden in the study cohorts based on the number of genes with RDVs

Supplementary Table 10. Effect of M1 Macrophages on survival in TCGA cases

List of Supplementary Data

Supplementary Data 1. List of genes with significant burden in multi-cancer and tissue-specific gene level burden analysis. Data in Supp_Data_1_genelevel.xlsx

Supplementary Data 2. The gene-set level burden (odds ratio (OR) and *p*-value) of rare, deleterious variant in Fanconi Anemia gene-set for multi-cancer and tissue-specific cancer. Data in Supp_Data_2_FAgenesetlevel.xlsx

Supplementary Data 3. The gene-set level burden (odds ratio (OR) and *p*-value) of rare, deleterious variant in CPD, DDR, SCD and *FA* gene-sets for pan-gastrointestinal, pan-kidney, pan-gynecological, and pan-squamous cancer types. Data in Supp_Data_3_Pan_geneset.xlsx

Supplementary Data 4. The gene-set level burden (odds ratio (OR) and *p*-value) of rare, deleterious variant for all gene-sets listed in Molecular Signatures Database (MSigDB) for multi-cancer. Data in Supp_Data_4_MSigDB.xlsx

Supplementary Data 5. Comparison of immune cell fractions (from CIBERSORT) based on germline RDV load in TCGA cases for different gene-sets. Data in Supp_Data_5_TME.xlsx

Supplementary Data 6. Comparison of gene expression of genes related to M1 Macrophages (LM22 matrix) based on germline RDV load in TCGA cases for different gene-sets. Data in Supp_Data_6_M1Macrophages_Genes.xlsx

Supplementary Data 7. Comparison of tumor mutational burden (TMB) based on germline RDV load in TCGA cases for different gene-sets. Data in Supp_Data_7_TMB.xlsx

Supplementary Data 8. List of ICD9/10 codes for identifying cancer patients. Data in Supp_Data_8_ICD9_10.xlsx

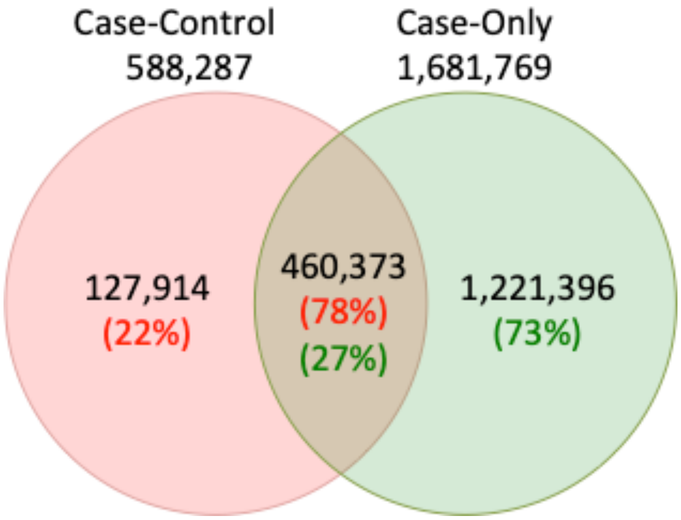

Supplementary Figure 1. Comparison of the variants identified in 6,275 TCGA cases from our case-control analysis and previous case-only analysis.

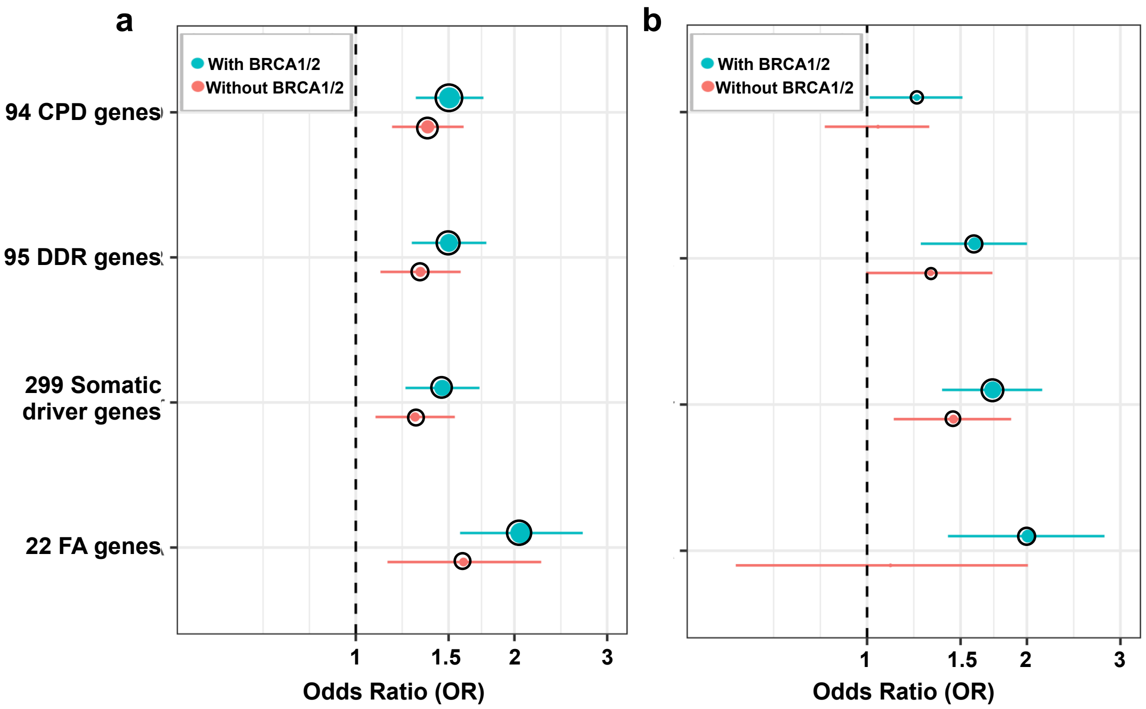

Supplementary Figure 2. Gene-set level rare, deleterious variant (RDV) burden in the study cohorts with and without BRCA1/2 RDVs. Gene-set level burden of rare, deleterious variants when using penalized logistic regression analysis (PLRA) (i) with BRCA1/2 RDVs (blue) and (ii) without BRCA1/2 RDVs (red). **a)** Discovery cohort; **b)** Validation cohort. The whiskers span the 95% confidence interval for OR values. The black circle outline indicates significant burden $p \leq 0.05$

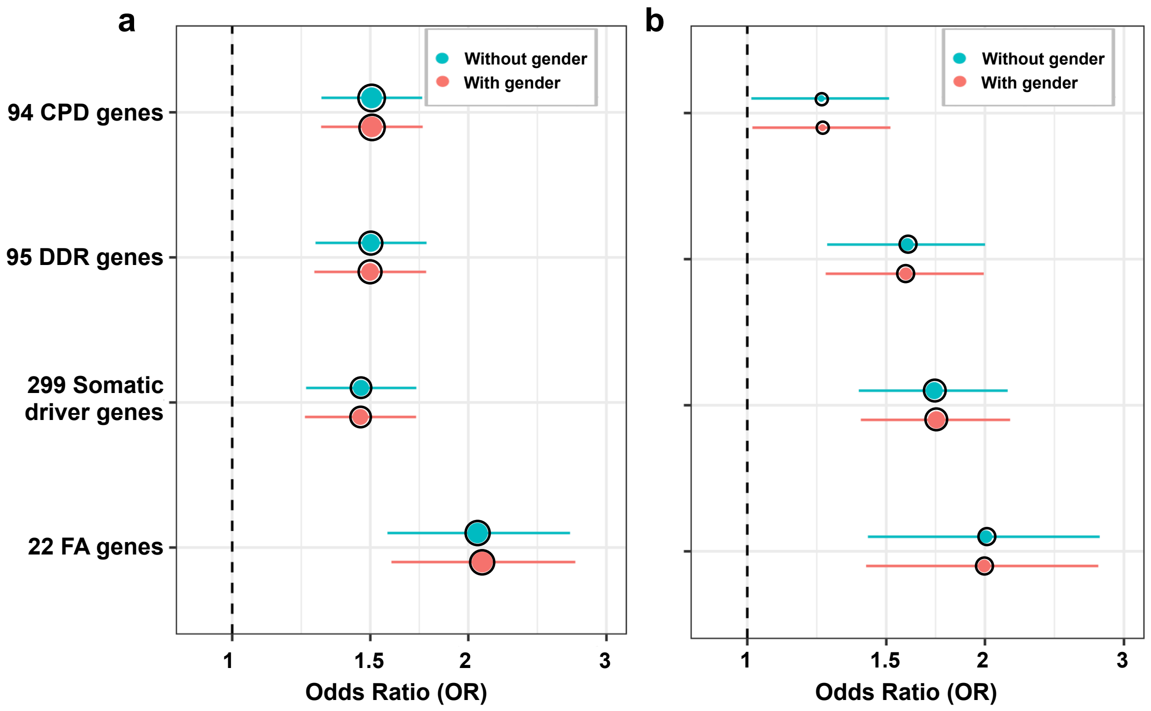

Supplementary Figure 3. Effect of gender on gene-set level burden of rare, deleterious variant in the study cohorts. Gene-set level burden of rare, deleterious variants when using penalized logistic regression analysis (PLRA) (i) without gender effect, by not using gender information as a covariate (blue) and (ii) with gender effect, by using the gender information as additional covariate (red). **a)** Discovery cohort; **b)** Validation cohort. The whiskers span the 95% confidence interval for OR values. The black circle outline indicates significant burden $p \leq 0.05$

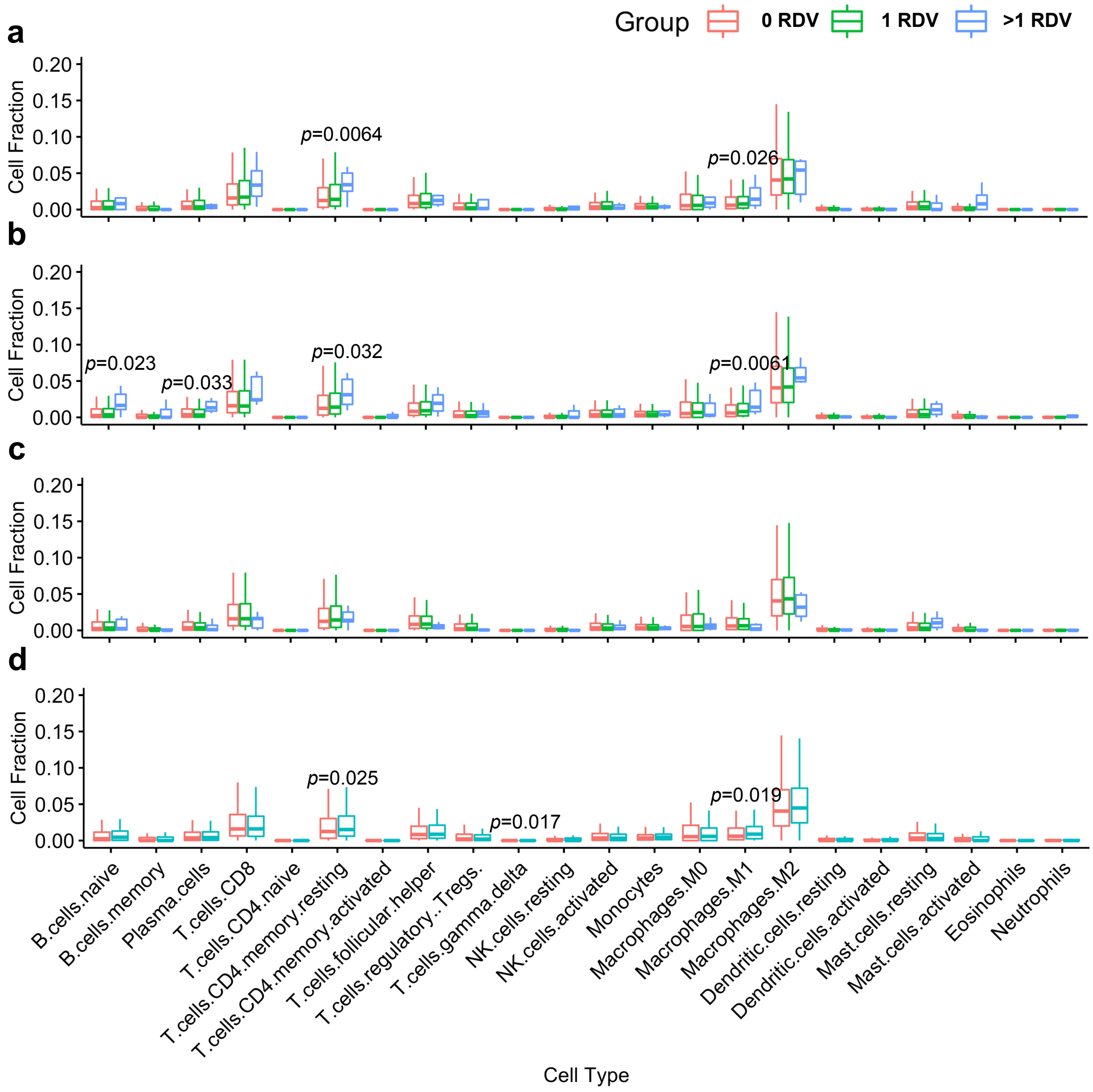

Supplementary Figure 4. Comparison of immune cell fractions (from CIBERSORT) based on germline RDV load in TCGA cases for different gene-sets. a) 94 Cancer predisposition genes **b)** 95 DNA damage repair genes **c)** 299 Somatic cancer driver genes **d)** 22 Fanconi Anemia genes. Significant *p* values from Kruskal-Wallis test are included in the figure. Boxplot elements: center line indicates median; box limits represent lower (25th percentile) and upper (75th percentile) quartiles; whiskers extend to 1.5 times the interquartile range.

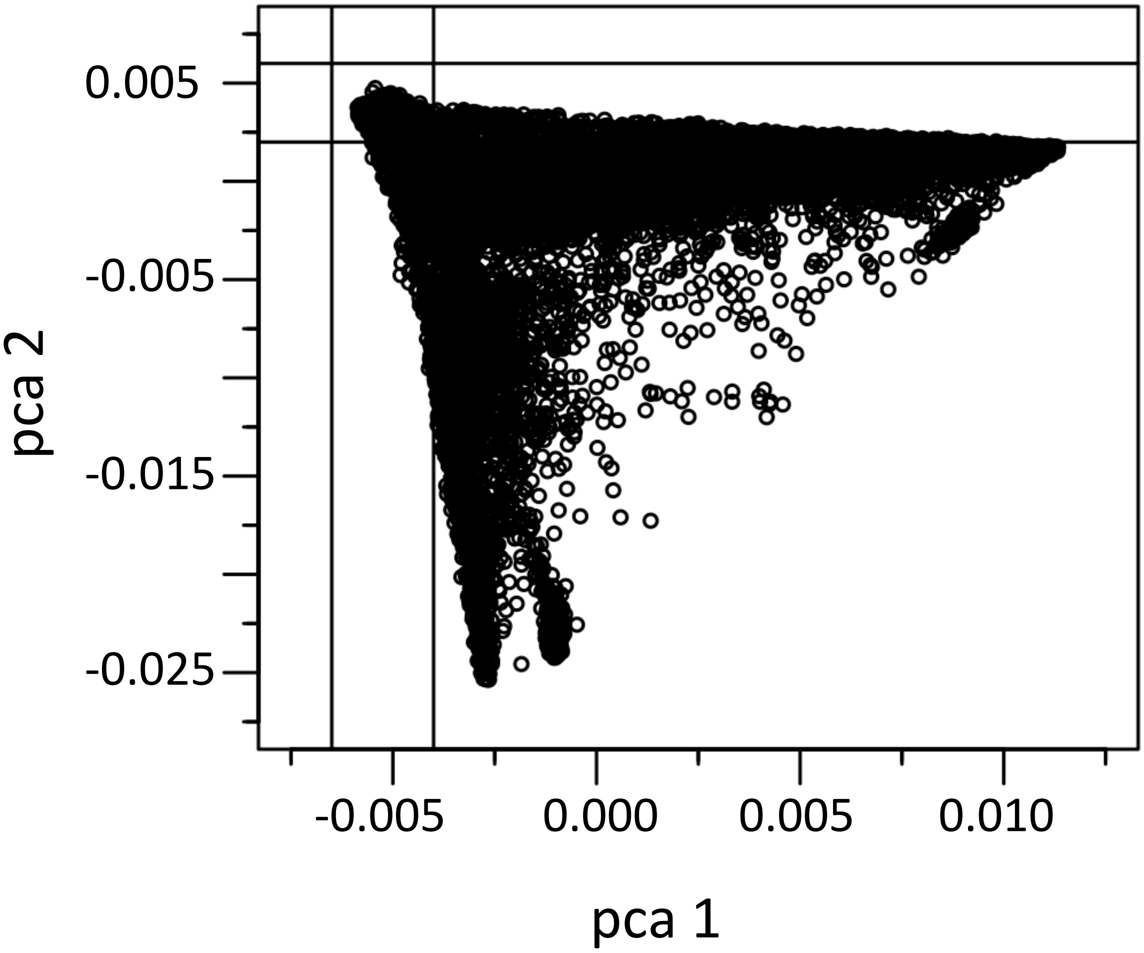

Supplementary Figure 5. Gating individuals of European ancestry in the BioMe cohort. 10,784 individuals of European ancestry were gated using the top two principal components.

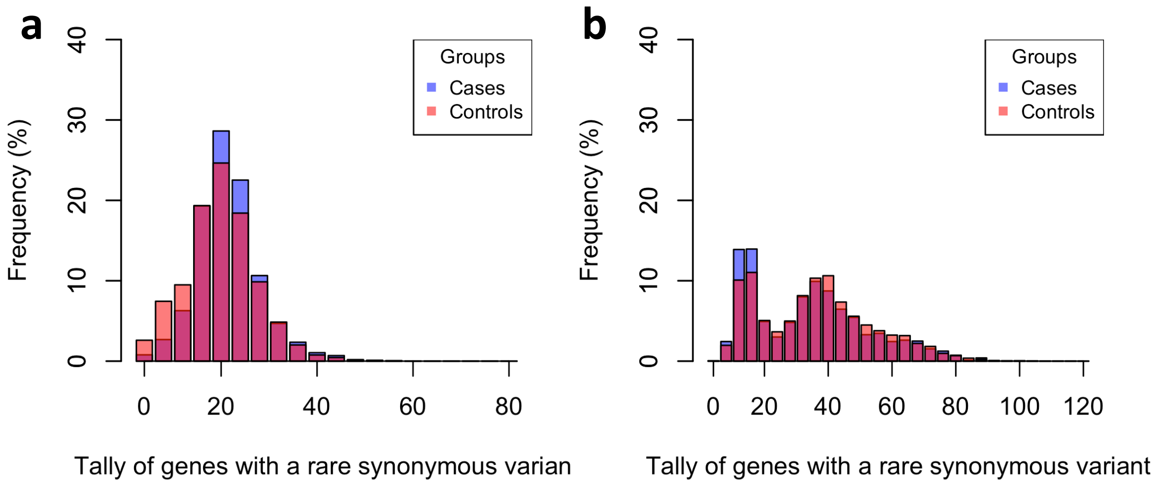

Supplementary Figure 6. Tally of genes with per-sample rare synonymous variants between cases and controls. **a)** Discovery cohort, Cases: average 19.98±6.7 genes, Controls: average 18.38±7.3 genes, Mann-Whitney U test *p*-value < 2.2e-16. **b)** Validation cohort, Cases: average 32.36±18.35 genes, Controls: average 34.60±17.87 genes, Mann-Whitney U test *p*-value = 2.75e-07.

Supplementary Table 1. Distribution of samples in TCGA (https://www.cancer.gov/tcga)

Sl.No	Cancer Type	# Cases before QC	# Cases after QC
1	Breast invasive carcinoma	848	622
2	Lung adenocarcinoma	546	463
3	Lung squamous cell carcinoma	494	443
4	Brain Lower Grade Glioma	508	426
5	Head and Neck squamous cell carcinoma	526	426
6	Skin Cutaneous Melanoma	453	424
7	Prostate adenocarcinoma	491	403
8	Thyroid carcinoma	487	338
9	Uterine Corpus Endometrial Carcinoma	480	331
10	Bladder Urothelial Carcinoma	400	305
11	Kidney renal clear cell carcinoma	367	279
12	Stomach adenocarcinoma	415	268
13	Colon adenocarcinoma	350	267
14	Glioblastoma multiforme	310	252
15	Ovarian serous cystadenocarcinoma	277	235
16	Kidney renal papillary cell carcinoma	280	193
17	Liver hepatocellular carcinoma	363	167
18	Cervical SqCC and endocervical adenocarcinoma	283	162
19	Rectum adenocarcinoma	107	99
20	Adrenocortical carcinoma	90	76
21	Acute Myeloid Leukemia	75	67
22	Kidney Chromophobe	66	56
23	Uterine Carcinosarcoma	57	42
24	Lymphoid Neoplasm Diffuse Large B-cell Lymphoma	48	27
	Total	8321	6371

Supplementary Table 2. dbGaP control cohort. Total number of samples in each dbGaP (http://www.ncbi.nlm.nih.gov/gap) study before and after sample QC.

dbGaP Study Accession	Study Name	Start Count	Final Count
phs000209	Multi-Ethnic Study of Atherosclerosis (MESA) Cohort	401	242
phs000276	STAMPEED: Northern Finland Birth Cohort 1966 (NFBC1966)	514	513
phs000296	NHLBI GO-ESP: Lung Cohorts Exome Sequencing Project (COPDGene)	290	280
phs000298	ARRA Autism Sequencing Collaboration	168	168
phs000424	Common Fund (CF) Genotype-Tissue Expression Project (GTEx)	478	380
phs000654	Genetic Analyses in Epileptic Encephalopathies	583	457
phs000687	Bulgarian Trio Sequencing Study to Identify de Novo Mutations in Schizophrenia	555	524
phs000806	Myocardial Infarction Genetics Exome Sequencing Consortium: Ottawa Heart Study	964	942
phs000876	Transdisciplinary Research Into Cancer of the Lung (TRICL) - Exome Plus Targeted Sequencing	591	551
phs000971	The ClinSeq Project: Piloting large-scale genome sequencing for research in genomic medicine	621	519
phs001000	Myocardial Infarction Genetics Exome Sequencing Consortium: U. of Leicester	1153	1022
phs001101	Myocardial Infarction Genetics Exome Sequencing Consortium: Malmo Diet and Cancer Study	1070	1049
**Total**		7388	6647

Supplementary Table 3. List of variants identified during germline variant calling in a case-control setting in the discovery cohort after sample and variant QC (6,371 cases and 6,647 controls)

	**Variant Type**	**Number of sites**
1	Exonic	935,912
2	Exonic; splicing	245
3	ncRNA_exonic; splicing	18
4	ncRNA_splicing	21
5	Splicing	5,413
		941,609

Supplementary Table 4. Count of variants identified in prior case-only approach, our case-control approach and TOPMed

Case-Only Approach	Case-Control Approach	TOPMed	Count
FALSE	TRUE	TRUE	63,364
TRUE	FALSE	TRUE	713,548
TRUE	TRUE	TRUE	311,040
TRUE	FALSE	FALSE	507,848
TRUE	TRUE	FALSE	149,333
FALSE	TRUE	FALSE	64,550

Supplementary Table 5. Comparison of the genotypes identified in prior case-only approach, our case-control approach and TOPMed

Set called in	SNV	Alt allele in TOPMed	Case-Control Approach			Case-only Approach		
			Wild-Type	Alt Allele	No call	Wild-Type	Alt Allele	No call
Both	TRUE	No	19,166,147,487	209,163,900	244,513,121		224,400,086	19,391,960,513
	TRUE	Yes	217,748,300	334,604,986	8,947,966		338,260,924	222,926,977
	FALSE	No	469,645,253	1,839,433	6,241,998	2	2,004,526	475,635,491
	FALSE	Yes	1,922,291	1,734,420	114,221	1	1,787,149	1,983,083
Just case-control approach	TRUE	No	538,054,583	2,957,801	7,786,930			
	TRUE	Yes	3,118,583	2,713,382	81,985			
	FALSE	No	12,324,896	83,372	237,873			
	FALSE	Yes	85,516	134,286	2,649			
Just case-only approach	TRUE	No					84,323,770	15,378,080,045
	TRUE	Yes					135,329,018	413,489,119
	FALSE	No				21	3,194,128	860,681,403
	FALSE	Yes				1	4,388,505	29,973,286

Supplementary Table 6. List of genes used in gene-set level burden analyses

	Gene Symbol
Gene-Set I: ***94 TruSight risk panel genes***	*AIP, ALK, APC, ATM, BAP1, BLM, BMPR1A, BRCA1, BRCA2, BRIP1, BUB1B, CDC73, CDH1, CDK4, CDKN1C, CDKN2A, CEBPA, CEP57, CHEK2, CYLD, DDB2, DICER1, DIS3L2, EGFR, EPCAM, ERCC2, ERCC3, ERCC4, BIVM-ERCC5, EXT1, EXT2, EZH2, FANCA, FANCB, FANCC, FANCD2, FANCE, FANCF, FANCG, FANCI, FANCL, FANCM, FH, FLCN, GATA2, GPC3, HNF1A, HRAS, KIT, MAX, MEN1, MET, MLH1, MSH2, MSH6, MUTYH, NBN, NF1, NF2, NSD1, PALB2, PHOX2B, PMS1, PMS2, PRF1, PRKAR1A, PTCH1, PTEN, RAD51C, RAD51D, RB1, RECQL4, RET, RHBDF2, RUNX1, SBDS, SDHAF2, SDHB, SDHC, SDHD, SLX4, SMAD4, SMARCB1, STK11, SUFU, TMEM127, TP53, TSC1, TSC2, VHL, WRN, WT1, XPA, XPC*
Gene-Set II: ***95 DNA repair genes***	*ALKBH2, ALKBH3, APEX1, APEX2, ATM, ATR, ATRIP, BAP1, BARD1, BIVM-ERCC5, BLM, BRCA1 (FANCS), BRCA2 (FANCD1), BRIP1 (FANCJ), CHEK1, CHEK2, CUL5, EME1, ERCC1, ERCC2, ERCC4 (FANCQ), ERCC6, EXO1, FAM175A, FANCA, FANCB, FANCC, FANCD2, FANCE, FANCF, FANCG, FANCI, FANCL, FANCM, FEN1, GEN1, LIG4, MAD2L2, MDC1, MGMT, MLH1, MLH3, MRE11A, MSH2, MSH3, MSH6, MUS81, NBN, NHEJ1, NUDT1, NUDT15, NUDT18, PALB2 (FANCN), PARP1, PMS1, PMS2, POLB, POLE, POLE3, POLL, POLM, POLN, POLQ, PRKDC, RAD50, RAD51 (FANCR), RAD51C (FANCO), RAD51D, RAD52, RBBP8, REV1, REV3L, RFWD3 (FANCW), RNMT, RRM1, RRM2, SHFM1, SHPRH, SLX1A, SLX4 (FANCP), TDG, TDP1, TOP3A, TOPBP1, TP53BP1, TREX1, UBE2T (FANCT), UNG, XPA, XPC, XRCC2 (FANCU), XRCC3, XRCC4, XRCC5, XRCC6*
Gene-Set III: ***299 cancer driver genes***	*ABL1, ACVR1, ACVR1B, ACVR2A, AJUBA, AKT1, ALB, ALK, AMER1, APC, APOB, AR, ARAF, ARHGAP35, ARID1A, ARID2, ARID5B, ASXL1, ASXL2, ATF7IP, ATM, ATR, ATRX, ATXN3, AXIN1, AXIN2, B2M, BAP1, BCL2, BCL2L11, BCOR, BRAF, BRCA1, BRCA2, BRD7, BTG2, CACNA1A, CARD11, CASP8, CBFB, CBWD3, CCND1, CD70, CD79B, CDH1, CDK12, CDK4, CDKN1A, CDKN1B, CDKN2A, CDKN2C, CEBPA, CHD3, CHD4, CHD8, CHEK2, CIC, CNBD1, COL5A1, CREB3L3, CREBBP, CSDE1, CTCF, CTNNB1, CTNND1, CUL1, CUL3, CYLD, CYSLTR2, DACH1, DAZAP1, DDX3X, DHX9, DIAPH2, DICER1, DMD, DNMT3A, EEF1A1, EEF2, EGFR, EGR3, EIF1AX, ELF3, EP300, EPAS1, EPHA2, EPHA3, ERBB2, ERBB3, ERBB4, ERCC2, ESR1, EZH2, FAM46D, FAT1, FBXW7, FGFR1, FGFR2, FGFR3, FLNA, FLT3, FOXA1, FOXA2, FOXQ1, FUBP1, GABRA6, GATA3, GNA11, GNA13, GNAQ, GNAS, GPS2, GRIN2D, GTF2I, H3F3A, H3F3C, HGF, HIST1H1C, HIST1H1E, HLA-A, HLA-B, HRAS, HUWE1, IDH1, IDH2, IL6ST, IL7R, INPPL1, IRF2, IRF6, JAK1, JAK2, JAK3, KANSL1, KDM5C, KDM6A, KEAP1, KEL, KIF1A, KIT, KLF5, KMT2A, KMT2B, KMT2C, KMT2D, KRAS, KRT222, LATS1, LATS2, LEMD2, LZTR1, MACF1, MAP2K1, MAP2K4, MAP3K1, MAP3K4, MAPK1, MAX, MECOM, MED12, MEN1, MET, MGA, MGMT, MLH1, MSH2, MSH3, MSH6, MTOR, MUC6, MYC, MYCN, MYD88, MYH9, NCOR1, NF1, NF2, NFE2L2, NIPBL, NOTCH1, NOTCH2,*

	NPM1, NRAS, NSD1, NUP133, NUP93, PAX5, PBRM1, PCBP1, PDGFRA, PDS5B, PGR, PHF6, PIK3CA, PIK3CB, PIK3CG, PIK3R1, PIK3R2, PIM1, PLCB4, PLCG1, PLXNB2, PMS1, PMS2, POLE, POLQ, POLRMT, PPM1D, PPP2R1A, PPP6C, PRKAR1A, PSIP1, PTCH1, PTEN, PTMA, PTPDC1, PTPN11, PTPRC, PTPRD, RAC1, RAD21, RAF1, RARA, RASA1, RB1, RBM10,, RET, RFC1, RHEB, RHOA, RHOB, RIT1, RNF111, RNF43, RPL22, RPL5, RPS6KA3, RQCD1, RRAS2, RUNX1, RXRA, SCAF4, SETBP1, SETD2, SF1, SF3B1, SIN3A, SMAD2, SMAD4, SMARCA1, SMARCA4, SMARCB1, SMC1A, SMC3, SOS1, SOX17, SOX9, SPOP, SPTA1, SPTAN1, SRSF2, STAG2, STK11, TAF1, TBL1XR1, TBX3, TCEB1, TCF12, TCF7L2, TET2, TGFBR2, TGIF1, THRAP3, TLR4, TMSB4X, TNFAIP3, TP53, TRAF3, TSC1, TSC2, TXNIP, U2AF1, UNCX, USP9X, VHL, WHSC1, WT1, XPO1, ZBTB20, ZBTB7B, ZC3H12A, ZCCHC12, ZFHX3, ZFP36L1, ZFP36L2, ZMYM2, ZMYM3, ZNF133, ZNF750
Gene-Set IV: ***22 Fanconi Anemia genes***	*FANCA, FANCB, FANCC, FANCD1 (BRCA2), FANCD2, FANCE, FANCF, FANCG, FANCI, FANCJ (BRIP1), FANCL, FANCM, FANCN (PALB2), FANCO (RAD51C), FANCP (SLX4), FANCQ (ERCC4), FANCR (RAD51), FANCS (BRCA1), FANCT (UBE2T), FANCU (XRCC2), FANCV (REV7), FANCW (RFWD3)*

Supplementary Table 7. Gene-set level rare, deleterious variant (RDV) burden in the study cohorts without BRCA1/2 RDVs

	TCGA-dbGaP Cohort		BioMe Cohort	
	**Cases (6,371)**	**Controls (6,647)**	**Cases (1,571)**	**Controls (6,200)**
	**92 CPD genes**			
# Variants	226	168	67	162
# Genes	55	46	33	49
# Unique individuals	392 (6.15%)	300 (4.51%)	101 (6.43%)	384 (6.19%)
OR (*p*-value) [95% CI]	1.37 (7.61e-05) [1.17-1.60]		1.05 (0.68) [0.83-1.31]	
	**93 DDR genes**			
# Variants	206	163	61	130
# Genes	39	34	27	33
# Unique individuals	300 (4.71%)	243 (3.66%)	71 (4.52%)	217 (3.50%)
OR (*p*-value) [95% CI]	1.33 (1.54e-03) [1.11-1.58]		1.32 (0.05) [1.00-1.72]	
	**297 Somatic Driver genes**			
# Variants	183	136	67	139
# Genes	57	50	38	57
# Unique individuals	307 (4.82%)	247 (3.72%)	85 (5.41%)	236 (3.81%)
OR (*p*-value) [95% CI]	1.30 (3.3e-03) [1.09-1.54]		1.45 (4.94e-03) [1.12-1.87]	
	**20 Fanconi Anemia genes**			
# Variants	67	40	11	39
# Genes	13	11	6	11
# Unique individuals	88 (1.38%)	59 (0.89%)	12 (0.76%)	46 (0.74%)
OR (*p*-value) [95% CI]	1.60 (5.41e-03) [1.15-2.25]		1.11 (0.75) [0.57-2.01]	

Supplementary Table 8: Distribution of males and females in the study cohorts

a). Discovery cohort

Sl.No	Cancer Type	Sample	Male	Female	Missing
1	Breast invasive carcinoma	622	6 (0.96%)	616 (99.04%)	0
2	Lung adenocarcinoma	463	196 (42.33%)	231 (49.89%)	36 (7.78%)
3	Lung squamous cell carcinoma	443	333 (75.17%)	110 (24.83%)	0
4	Brain Lower Grade Glioma	426	237 (55.63%)	188 (44.13%)	1 (0.23%)
5	Head and Neck squamous cell carcinoma	426	305 (71.60%)	121 (28.40%)	0
6	Skin Cutaneous Melanoma	424	258 (60.85%)	166 (39.15%)	0
7	Prostate adenocarcinoma	403	403 (100%)	0	0
8	Thyroid carcinoma	338	92 (27.22%)	246 (72.78%)	0
9	Uterine Corpus Endometrial Carcinoma	331	0	328 (99.09%)	3 (0.91%)
10	Bladder Urothelial Carcinoma	305	228 (74.75%)	77 (25.25%)	0
11	Kidney renal clear cell carcinoma	279	183 (65.59%)	96 (34.41%)	0
12	Stomach adenocarcinoma	268	159 (59.33%)	109 (40.67%)	0
13	Colon adenocarcinoma	267	144 (53.93%)	123 (46.07%)	0
14	Glioblastoma multiforme	252	159 (63.10%)	92 (36.51%)	1 (0.40%)
15	Ovarian serous cystadenocarcinoma	235	0	235 (100%)	0
16	Kidney renal papillary cell carcinoma	193	146 (75.65%)	47 (24.35%)	0
17	Liver hepatocellular carcinoma	167	92 (55.09%)	75 (44.91%)	0
18	Cervical SqCC and endocervical adenocarcinoma	162	0	162 (100%)	0
19	Rectum adenocarcinoma	99	52 (52.53%)	46 (46.46%)	1 (1.01%)
20	Adrenocortical carcinoma	76	28 (36.84%)	48 (63.16%)	0
21	Acute Myeloid Leukemia	67	34 (50.75%)	33 (49.25%)	0
22	Kidney Chromophobe	56	33 (58.93%)	23 (41.07%)	0
23	Uterine Carcinosarcoma	42	0	42 (100%)	0
24	Lymphoid Neoplasm Diffuse Large B-cell Lymphoma	27	11 (40.74%)	16 (59.26%)	0
	**Total cases**	6371	3099 (48.64%)	3230 (50.70%)	42 (0.66%)

	**Total controls**	6647	4034 (60.69%)	2610 (39.27%)	3 (0.05%)

b). Validation cohort

	Male	Female
Cases (1,571)	690 (43.92%)	881 (56.08%)
Controls (6,200)	3150 (50.81%)	3050 (49.19%)

Supplementary Table 9. Gene-set level rare, deleterious variant (RDV) burden in the study cohorts based on the number of genes with RDVs

	TCGA-dbGaP Cohort		BioMe Cohort	
	Cases (6,371)	Controls (6,647)	Cases (1,571)	Controls (6,200)
	**94 Cancer predisposition genes**			
# Indiv. without RDVs	5907	6321	1435	5761
# Indiv. with RDVs in 1 gene	452	323	128	426
# Indiv. with RDVs in ≥ 2 genes	12	3	8	13
Comparing Indv with RDV in 1vs0 OR (*p*-value) [95% CI]	1.48 (1.68e-07) [1.28-1.72]		1.20 (0.08) [0.98-1.47]	
Comparing Indv with RDV in ≥2vs0 OR (*p*-value) [95% CI]	3.31 (0.03) [1.10-13.02]		2.55 (0.04) [1.03-5.94]	
	**95 DNA damage repair genes**			
# Indiv. without RDVs	5997	6378	1463	5927
# Indiv. with RDVs in 1 gene	367	269	103	268
# Indiv. with RDVs in ≥ 2 genes	7	0	5	5
Comparing Indiv with RDV in 1vs0 OR (*p*-value) [95% CI]	1.47 (2.80e-06) [1.25-1.74]		1.55 (3.75e-04) [1.22-1.95]	
Comparing Indiv with RDV in ≥2vs0 OR (*p*-value) [95% CI]	15.13 (0.007) [1.82-1968.26]		3.96 (0.03) [1.17-13.41]	
	**299 Somatic cancer driver genes**			
# Indiv. without RDVs	5994	6375	1446	5906
# Indiv. with RDVs in 1 gene	366	267	123	288
# Indiv. with RDVs in ≥ 2 genes	11	5	2	6
Comparing Indiv with RDV in 1vs0 OR (*p*-value) [95% CI]	1.44 (9.56e-06) [1.23-1.70]		1.73 (2.01e-06) [1.39-2.15]	
Comparing Indiv with RDV in ≥2vs0 OR (*p*-value) [95% CI]	2.19 (0.12) [0.82-6.63]		1.56 (0.56) [0.29-6.12]	
	**22 Fanconi Anemia genes**			
# Indiv. without RDVs	6208	6562	1519	6097
# Indiv. with RDVs in 1 gene	163	85	51	101
# Indiv. with RDVs in ≥ 2 genes	0	0	1	2
Comparing Indiv with RDV in 1vs0 OR (*p*-value) [95% CI]	2.05 (6.14e-08) [1.58-2.70]		2.01 (1.24e-04) [1.42-2.80]	
Comparing Indiv with RDV in ≥2vs0 OR (*p*-value) [95% CI]	-		2.38 (0.42) [0.22-17.93]	

Supplementary Table 10. Effect of M1 Macrophages on survival in TCGA cases

	Cancer Type	Number Indiv	Cox_Coeff	Cox_p
1	Breast invasive carcinoma	606	-8.00	0.301
2	Lung adenocarcinoma	415	5.23	0.352
3	Lung squamous cell carcinoma	439	-1.98	0.699
4	**Brain lower grade glioma**	**426**	**43.17**	**0.018**
5	Head and neck squamous cell carcinoma	419	4.29	0.196
6	Skin cutaneous melanoma	422	-9.60	0.065
7	Prostate adenocarcinoma	401	-12.18	0.872
8	Thyroid carcinoma	337	-16.35	0.725
9	Uterine Corpus Endometrial Carcinoma	324	0.86	0.968
10	Bladder Urothelial Carcinoma	299	-1.46	0.794
11	**Kidney renal clear cell carcinoma**	**273**	**15.78**	**0.034**
12	Stomach adenocarcinoma	246	4.50	0.641
13	Colon adenocarcinoma	255	-17.47	0.270
14	Glioblastoma multiforme	99	7.52	0.578
15	Ovarian serous cystadenocarcinoma	181	4.43	0.783
16	**Kidney renal papillary cell carcinoma**	**192**	**56.41**	**0.017**
17	Liver hepatocellular carcinoma	164	-1.62	0.912
18	Cervical SqCC and endocervical adenocarcinoma	162	-12.85	0.240
19	Rectum adenocarcinoma	94	-163.52	0.321
20	Adrenocortical carcinoma	65	50.67	0.116
21	Kidney chromophobe	56	51.72	0.573
22	Uterine carcinosarcoma	42	22.86	0.346
