# Supplementary material for: Germline rare deleterious variant load alters cancer risk, age of onset and tumor characteristics
Source: NPJ Precis Oncol. 2023 Jan 27;7:13. doi: 10.1038/s41698-023-00354-3 (PMC9883433; doi:10.1038/s41698-023-00354-3)
Supplement: Supplementary file 10 — REPORTING SUMMARY [file 41698_2023_354_MOESM10_ESM.pdf]

nature portfolio

Corresponding author(s): Zeynep H. Gümüş

Last updated by author(s): Nov 21, 2022

Reporting Summary

Nature Portfolio wishes to improve the reproducibility of the work that we publish. This form provides structure for consistency and transparency in reporting. For further information on Nature Portfolio policies, see our Editorial Policies and the Editorial Policy Checklist.

Statistics

For all statistical analyses, confirm that the following items are present in the figure legend, table legend, main text, or Methods section.

n/a	Confirmed	
☐	☒	The exact sample size (n) for each experimental group/condition, given as a discrete number and unit of measurement
☒	☐	A statement on whether measurements were taken from distinct samples or whether the same sample was measured repeatedly
☐	☒	The statistical test(s) used AND whether they are one- or two-sided<br>*Only common tests should be described solely by name; describe more complex techniques in the Methods section.*
☐	☒	A description of all covariates tested
☐	☒	A description of any assumptions or corrections, such as tests of normality and adjustment for multiple comparisons
☐	☒	A full description of the statistical parameters including central tendency (e.g. means) or other basic estimates (e.g. regression coefficient) AND variation (e.g. standard deviation) or associated estimates of uncertainty (e.g. confidence intervals)
☐	☒	For null hypothesis testing, the test statistic (e.g. F, t, r) with confidence intervals, effect sizes, degrees of freedom and P value noted<br>*Give P values as exact values whenever suitable.*
☒	☐	For Bayesian analysis, information on the choice of priors and Markov chain Monte Carlo settings
☒	☐	For hierarchical and complex designs, identification of the appropriate level for tests and full reporting of outcomes
☒	☐	Estimates of effect sizes (e.g. Cohen's d, Pearson's r), indicating how they were calculated

Our web collection on statistics for biologists contains articles on many of the points above.

Software and code

Policy information about availability of computer code

Data collection	No software was used
Data analysis	Picard (1.07), KING software (1.4), EIGENSOFT (5.0.1), Annovar (2018Apr16)

For manuscripts utilizing custom algorithms or software that are central to the research but not yet described in published literature, software must be made available to editors and reviewers. We strongly encourage code deposition in a community repository (e.g. GitHub). See the Nature Portfolio guidelines for submitting code & software for further information.

Data

Policy information about availability of data

All manuscripts must include a data availability statement. This statement should provide the following information, where applicable:

- Accession codes, unique identifiers, or web links for publicly available datasets
- A description of any restrictions on data availability
- For clinical datasets or third party data, please ensure that the statement adheres to our policy

The results here are in part based upon data generated by the TCGA Research Network (https://www.cancer.gov/tcga) and datasets from dbGaP (http://www.ncbi.nlm.nih.gov/gap) through dbGaP accession numbers phs000209, phs000276, phs000296, phs000298, phs000424, phs000654, phs000687, phs000806, phs000876, phs000971, phs001000, phs001101.

Human research participants

Policy information about studies involving human research participants and Sex and Gender in Research.

Reporting on sex and gender	We performed gender-based analyses (based on self-reported gender information).
Population characteristics	The population characteristics such gender, age and smoking history are available.
Recruitment	Not applicable
Ethics oversight	Not applicable

Note that full information on the approval of the study protocol must also be provided in the manuscript.

Field-specific reporting

Please select the one below that is the best fit for your research. If you are not sure, read the appropriate sections before making your selection.

☒ Life sciences ☐ Behavioural & social sciences ☐ Ecological, evolutionary & environmental sciences

For a reference copy of the document with all sections, see nature.com/documents/nr-reporting-summary-flat.pdf

Life sciences study design

All studies must disclose on these points even when the disclosure is negative.

Sample size	The sample size was based on the number of individuals in the underlying cohort studies.
Data exclusions	We included only samples for which over 75% of the exome was callable and there was no evidence of contamination. We also removed samples with 15% or more missing genotype data.
Replication	In this study, the results identified in the discovery cohort were replicated in an independent validation cohort.
Randomization	No randomization was used in this case-control study. We used the number of genes with rare synonymous variants as a covariate for each individual to adjust for background variation. Additionally, we used the first two principal components covariates in the discovery cohort to adjust for population difference. We also included gender as a covariate when applicable.
Blinding	The investigators were not blinded in the data analysis. The analysis was carried out in case-control setting, so the cases and controls were identified in the beginning of the analysis.

Reporting for specific materials, systems and methods

We require information from authors about some types of materials, experimental systems and methods used in many studies. Here, indicate whether each material, system or method listed is relevant to your study. If you are not sure if a list item applies to your research, read the appropriate section before selecting a response.

Materials & experimental systems

n/a	Involved in the study	
☒	☐	Antibodies
☒	☐	Eukaryotic cell lines
☒	☐	Palaeontology and archaeology
☒	☐	Animals and other organisms
☒	☐	Clinical data
☒	☐	Dual use research of concern

Methods

n/a	Involved in the study	
☒	☐	ChIP-seq
☒	☐	Flow cytometry
☒	☐	MRI-based neuroimaging
